# Tetrel Bonds with π-Electrons Acting as Lewis Bases—Theoretical Results and Experimental Evidences

**DOI:** 10.3390/molecules23051183

**Published:** 2018-05-15

**Authors:** Sławomir J. Grabowski

**Affiliations:** 1 Faculty of Chemistry, University of the Basque Country and Donostia International Physics Center (DIPC), P.K. 1072, 20080 Donostia, Spain; s.grabowski@ikerbasque.org; Tel.: +34-943-01-5477; 2 IKERBASQUE, Basque Foundation for Science, 48011 Bilbao, Spain

**Keywords:** electron charge shifts, tetrel bond, hydrogen bond, π-electrons as Lewis bases, σ-hole

## Abstract

MP2/aug-cc-pVTZ calculations were carried out for the ZFH_3_-B complexes (Z = C, Si, Ge, Sn and Pb; B = C_2_H_2_, C_2_H_4_, C_6_H_6_ and C_5_H_5_^-^; relativistic effects were taken into account for Ge, Sn and Pb elements). These calculations are supported by other approaches; the decomposition of the energy of interaction, Quantum Theory of Atoms in Molecules (QTAIM) and Natural Bond Orbital (NBO) method. The results show that tetrel bonds with π-electrons as Lewis bases are classified as Z···C links between single centers (C is an atom of the π-electron system) or as Z···π interactions where F‒Z bond is directed to the mid-point (or nearly so) of the CC bond of the Lewis base. The analogous systems with Z···C/π interactions were found in the Cambridge Structural Database (CSD). It was found that the strength of interaction increases with the increase of the atomic number of the tetrel element and that for heavier tetrel elements the ZFH_3_ tetrahedral structure is more deformed towards the structure with the planar ZH_3_ fragment. The results of calculations show that the tetrel bond is sometimes accompanied by the Z-H···C hydrogen bond or even sometimes the ZFH_3_-B complexes are linked only by the hydrogen bond interaction.

## 1. Introduction

The tetrel bond is a Lewis acid—Lewis base interaction that may play an important role in some chemical and biological processes [1]; for example, it may be considered as a preliminary stage of the S_N_2 reaction [2]. This interaction was classified as the σ-hole bond by Politzer and coworkers since it may be defined as an interaction between the 14th Group element acting as the Lewis acid centre through its σ-hole and a region that is rich of the electron density by a lone electron pair, π-electron system etc. [3,4]. The σ-hole is usually located in this case in the elongation of the covalent bond to the tetrel centre and it is often characterized by the positive electrostatic potential (EP) [3,4]. It seems that first time the tetrel bond was analyzed in terms of the σ-hole concept in the SiF_4_ complexes with amines [5]. In spite of the fact that the term “tetrel bond” appeared recently [6] and also this interaction was classified as the σ-hole bond in the last decade [3,4,5] it was analyzed in earlier studies. For example, the SiF_4_···NH_3_ and SiF_4_···(NH_3_)_2_ complexes were analyzed theoretically since ab initio MO calculations were performed with the use of STO-3G and STO-6G basis sets [7], another study that is more complex concerns a large sample of complexes of silicon derivatives with electron-rich groups [8]. Both latter studies were performed before the proposition of the σ-hole concept [9,10] and the introduction of the tetrel bond term [6]. 

One can mention other studies on tetrel bonds as for example that one where this interaction was analyzed as a preliminary stage of the S_N_2 reaction [11], the theoretical analysis of structural and energetic properties of acetonitrile complexes with the 14 Group tetrahalides [12]; a study on the Lewis acid carbon center, the corresponding interaction was labeled as the carbon bond and it was compared with the hydrogen bond [13]; it is worth mentioning that the carbon bond is a sub-class of the tetrel bond interactions [2,11]. The tetrel-hydride interaction is another sub-class of tetrel bonds where the negatively charged H-atom plays the role of Lewis base center [14].

There are other, more recent studies on this kind of interaction; only few are mentioned here; the analysis of factors which influence the strength of tetrel bonds [15], the analysis of mechanisms of S_N_2 reactions, among them at the C centre [16], the role of tetrel bonds in the crystal structures’ stabilization [17], the theoretical analysis of the H-Si···N and F-Si···N linear or nearly so arrangements [18], comparison of neutral and charge assisted tetrel bonds [19], the geometry deformations of monomers linked by tetrel bond [20] or the balance between the attractive forces of tetrel interactions and the steric repulsions in crystal structures [21]. 

One may cite numerous other examples since the number of studies on this kind of interaction has increased rapidly. However, it seems that there are no systematic and extensive studies on the tetrel bonds with π-electrons playing a role of Lewis bases or at most they are very rare and they are not a main goal of investigations. For example, very recently, various σ-hole bonds were analyzed with the use of few theoretical approaches: halogen, chalcogen, pnicogen and tetrel bonds were compared [22]; three different types of Lewis bases were considered there, neutral species (NH_3_), anion (Cl^−^) and the π-electron system (C_2_H_2_). Hence two tetrel bonded complexes with acetylene molecule playing a role of the Lewis base were analyzed there among various other complexes. These are the SiFH_3_···C_2_H_2_ and GeFH_3_···C_2_H_2_ complexes [22]. 

The other issue that is not analyzed so frequently concerns the tetrel bond interactions with the heavier tetrel elements, such analyses are rather rare and mainly concern germanium species. There are more experimental studies on tetrel bonds with heavier tetrel elements playing a role of the Lewis acid centers, however only sometimes such experimental analyses are supported by theoretical results [23]. One of examples where heavier tetrel elements were considered in tetrel bonds is a study on the SnF_4_ and PbF_4_ complexes with NH_3_ and HCN that play a role of Lewis bases through the nitrogen centre [24]. A theoretical study analogous to the latter one was performed on the lighter tetrel species since the complexes of CF_4_, SiF_4_ and GeF_4_ Lewis acid units with NH_3_ and AsH_3_ Lewis bases were analyzed [25]. 

Returning to the π-electron species—numerous theoretical and experimental studies on interactions where such systems play a role of Lewis bases may be mentioned. These are mainly those studies that concern hydrogen bonded systems [26]. However other Lewis acid—Lewis base interactions with π-electron donors were analyzed very often [27,28,29,30]. One can even mention the triel bonds between the boron or aluminium Lewis acid center and acetylene or ethylene [31,32] or the recent study where the multivalent halogen centers act as the Lewis acids [33].

The aim of this study is an analysis of the tetrel bonds in complexes of ZFH_3_ species, where Z labels the following centers; C, Si, Ge, Sn and Pb, thus light and heavy tetrels are taken into account; the acetylene, ethylene, benzene and cyclopentadienyl anion were chosen as the π-electron moieties acting as the Lewis bases. Different theoretical techniques are applied here to deepen the understanding of the nature of these tetrel bond interactions; i.e., the Quantum Theory of Atoms in Molecules (QTAIM) [34], Natural Bond Orbital (NBO) approach [35], the decomposition of the energy of interaction [36,37] as well as the analysis of the electrostatic potential (EP) distribution [38]. The short descriptions of the theoretical approaches applied here are included in the section that concerns the computational details.

## 2. Results and Discussion

### 2.1. Energetic and Geometric Parameters

**Figure 1:**
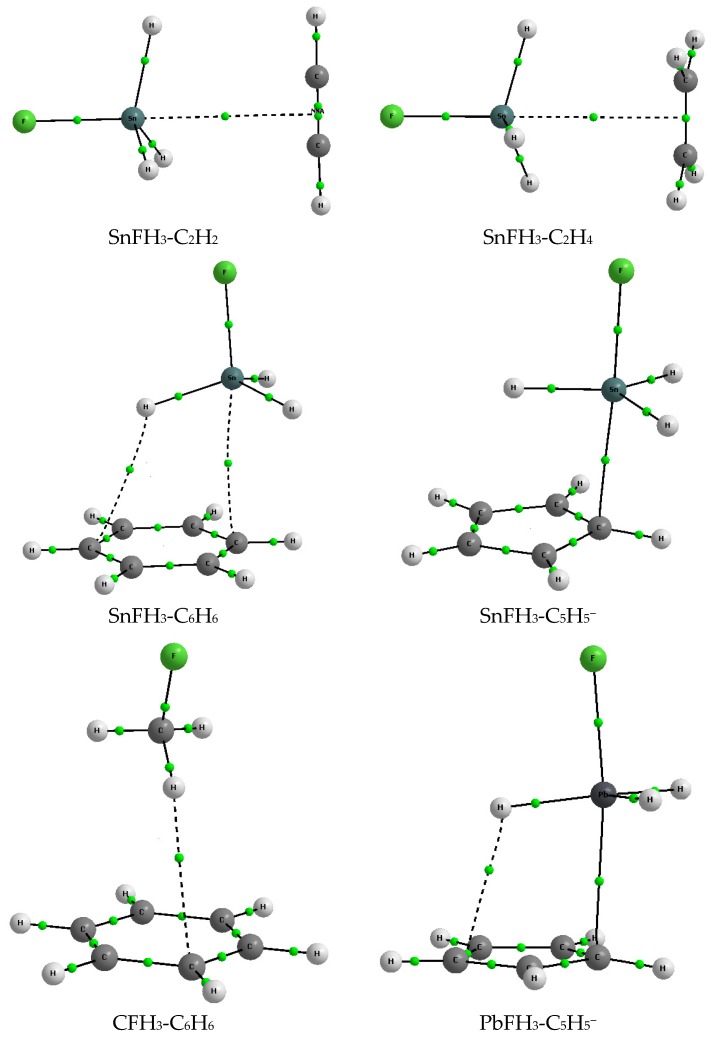
The molecular graphs of the selected complexes analyzed here; big circles—attractors, small green circles—BCPs, the nonnuclear attractor (NNA) is located (small red circle) between two BCPs of the CC bond in a case of the SnFH_3_-C_2_H_2_ complex.

Figure 1 presents examples of complexes analyzed here. All kinds of Lewis bases that are considered are shown in selected examples of the figure. The molecular graphs are presented since they reflect geometry of species analyzed. However these graphs are discussed further here in the section on QTAIM results.

The energetic parameters of analyzed complexes, among them, the binding and interaction energies corrected for BSSE, E_bin_ and E_int_, respectively, are included in Table 1. One can see much stronger interactions, i.e., greater –E_int_ and –E_bin_ values, for complexes with the cyclopentadienyl anion, than for complexes with the other Lewis base units. This may be explained since the complexes of C_5_H_5_^−^ anion that are linked through the tetrel bonds are assisted by negative charge; the latter anion is much stronger base than the remaining species chosen here. The following tendencies are also observed here, and it does not depend on the choice of –E_int_ or –E_bin_ value for the discussion; the strength of interaction for the same Lewis base increases in the following order of the tetrel center C < Si < Ge < Sn ≅ Pb. It was observed earlier for tetrel bond interactions [3,4] and it was explained by the increase of the electrostatic part of the energy of interaction since the electrostatic potential (EP) at the tetrel σ-hole increases with the increase of the atomic number [3,4]. The calculations performed here show the EP of tetrel σ-hole of the ZFH_3_ species equal to 0.033; 0.062; 0.068; 0.081; 0.080 au for C, Si, Ge, Sn and Pb centers, respectively (0.001 au electron density surfaces were chosen). The EP values for tin and lead centers are almost equal one to each other. This is why for complexes analyzed here similar Lewis acid properties are observed for these centers; however the strongest interaction is observed for the SnFH_3_-C_5_H_5_^−^ complex if the interaction energy is considered while if the binding energy is taken into account thus it is the PbFH_3_-C_5_H_5_^−^ complex. 

If the Lewis acid unit is the same thus the interaction strength increases in the following order C_2_H_2_ < C_2_H_4_ < C_6_H_6_ < C_5_H_5_^−^. One can also see that the –E_int_ or –E_bin_ values do not exceed 6 kcal/mol for all complexes of acetylene, ethylene and benzene while they are much greater in a case of complexes with cyclopentadienyl, especially large values are observed for the above-mentioned tin and lead complexes.

The BSSE corrections are greater for stronger interactions, especially large values are observed for interactions in cyclopentadienyl complexes. The deformation energy, E_def_, is a parameter that is related to geometrical changes of the interacting systems. For example, in a case of the strong A-H···B hydrogen bonds, the complexation often leads to the meaningful elongation of the A‒H proton donating bond that results in the greater E_def_ values [39]. Steric effects are very important for tetrel bonded species [11,21] since the tetrel center, often characterized by the sp^3^ hybridization and surrounded by four substituents (like for the systems considered here) is hardly available for the Lewis base (nucleophilic attack). Thus the tetrel-base link should cause greater deformations connected with the increase of the availability of the tetrel center. In other words the ZFH_3_ tetrahedral system should be closer to the trigonal bipyramid in the ZFH_3__‒_B complex with the ZH_3_ part being closer to planarity. For complexes of acetylene, ethylene and benzene E_def_ does not exceed 0.3 kcal/mol indicating negligible changes of geometry resulting from complexation. However for the C_5_H_5_^−^ complexes, if one excludes the CFH_3_-C_5_H_5_^−^ complex with this energy amounting only 0.3 kcal/mol, E_def_ is close to 10 kcal/mol or even exceeds this value. This is in agreement with changes of geometry; one can see (Figure 1) the ZH_3_ part close to planarity and the F-Z···C arrangement close to linearity for two complexes presented; SnFH_3_-C_5_H_5_^−^ and PbFH_3_-C_5_H_5_^−^. 

The above-presented EP values at the Z-tetrel centre concern the σ-hole that occurs in the extension of F‒Z bonds. For the ZFH_3_ species analyzed here, similarly as for other sp^3^ hybridized tetrel centers four σ-holes located in extensions of covalent bonds to Z-center occur. However the electronegative F-substituent enhances F‒Z σ-hole [3,4] that results in greater positive EP values than those of other H‒Z σ-holes. For example, for the SnFH_3_ molecule the EP value at the F‒Sn σ-hole is equal to +0.081 au while this value for the H‒Sn σ-hole amounts +0.048 au. For the clarity of the results’ presentation only interactions of the F‒Z σ-hole are considered here; it means that the F‒Z σ-hole is directed to the π-electron system in the configurations analyzed. 

Table 1 presents the Lewis acid—Lewis base distances, for each of complexes the shortest Z···C contact was chosen. One can see that these distances are usually greater than 3 Å, only for the C_5_H_5_^−^ complexes where stronger interactions are observed such distances amount ~2.5 Å (except of the CFH_3_-C_5_H_5_^−^ complex where this distance is equal to ~3.2 Å). It was pointed out in numerous studies that the distance between interacting units is roughly related to the strength of interaction, this was observed for the hydrogen bonded complexes [40] but it seems that such dependence occurs also for other types of interactions [2]. It is often stated in various studies that the sum of van der Waals radii of two atoms being in contact roughly indicates at which distance a significant so-called noncovalent interaction begins [41]. Table 1 presents how the Z···C distances are related to the corresponding sum of Z and C van der Waals radii. These distances are greater than the corresponding sum for carbon complexes (Z = C) and for the SiFH_3_-C_2_H_2_ complex while for the remaining ones these distances are lower than the van der Waals sum; the following van der Waals radii were applied here, H—1.2 Å [42], C—1.7 Å, Si—2.1 Å, Ge—2.1 Å, Sn—2.25 Å and Pb—2.3 Å [43]. One can see that for the strongest interactions in the C_5_H_5_^-^ complexes the Z···C distance is almost by 1 Å lower than the sum of van der Waals radii. If one considers only the Z···C distances as a measure of the strength of interaction thus the interactions in the CFH_3_ complexes are very weak and one may contest even their stabilizing nature. 

**Figure 2:**
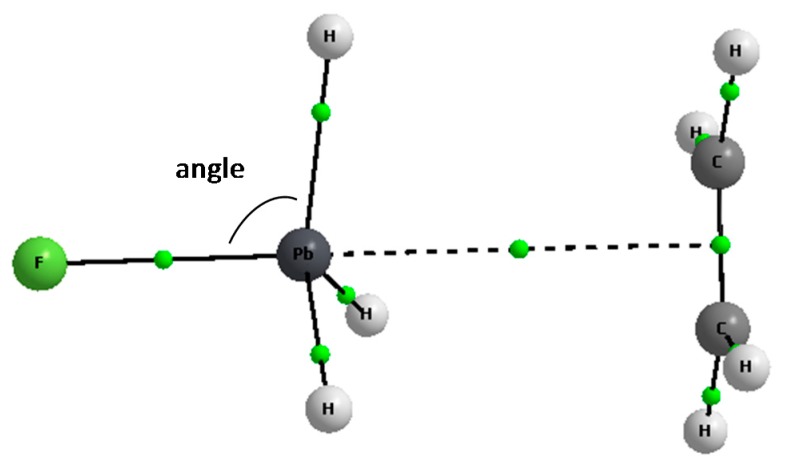
The definition of the angle expressing the change of the tetrahedral structure into the structure being closer to the trigonal bipyramid.

Table 2 presents geometrical parameters related to the changes resulting from complexation; one of them is a percentage elongation of the Z‒F bond related to the corresponding isolated ZFH_3_ species that is not involved in the tetrel bond. One can see that these elongations correspond to the deformation energies, the greatest values are observed for the cyclopentadienyl complexes. For the species analyzed here three F‒Z‒H angles in the Lewis acid unit are very close one to each other; however for each complex considered its average value is considered in the further discussions; the latter angle is defined in Figure 2. For the isolated ZFH_3_ species this tetrahedral angle, labeled as α_iso_, amounts from 101.4° for PbFH_3_ to 108.8° for CFH_3_. It decreases in the complex to α_comp_ (up to 90° corresponding to the planar ZH_3_ system in the trigonal bipyramid structure). The angle decrease values, [(α_iso_ − α_comp_)/α_iso_] × 100%, are presented in Table 2. They correspond to the deformation energies discussed earlier here as well as to the elongations of the Z‒F bonds since the greatest decreases are observed for the strongest interactions. The Z‒F bond elongations result from the π_CC_ → σ_ZF_^*^ and σ_CH_ → σ_ZF_^*^ overlaps; Table 2 shows the orbital-orbital NBO energies corresponding to these overlaps; E_NBO_^1^ is a sum of all energies of such interactions for the F−C bond considered. Similarly the E_NBO_^2^ energy summarizes all π_CC_ → σ_ZH_^*^ and σ_CH_ → σ_ZH_^*^ overlaps, however in this case the whole ZFH_3_ species is considered for one E_NBO_^2^ value (i.e., three C‒H bonds). One can see (Table 2) that both E_NBO_^1^ and E_NBO_^2^ values are much greater for the C_5_H_5_^−^ complexes than for other ones.

Table 2 shows the electron charge transfer values from the Lewis base to the Lewis acid unit, these transfers are especially great for the ZFH_3_-C_5_H_5_^−^ complexes, except of the CFH_3_-C_5_H_5_^−^ one. Such electron charge redistributions resulting from complexation are usually great for those complexes where the geometry deformations are important [2]; this is observed also here. The charge of the Z-central tetrel atom is shown in Table 2; one can see that this charge decreases (is “more negative”) in complexes in comparison with the corresponding isolated ZFH_3_ species. This is in opposite to the A-H···B hydrogen bonded systems where the complexation usually results in the increase of the positive charge of the central H-atom [35].

### 2.2. Nature of Interactions—Decomposition of Interaction Energy

Table 3 presents terms of the energy of interaction resulting from the Ziegler and Rauk decomposition scheme [36,37] (see the Computational Details section).

One can see that only for the CFH_3_ complexes with acetylene, ethylene and benzene the dispersion term, ΔE_disp_, is the most important attractive one. This is typical for weak van der Waals interactions where attractive interaction energy terms related to charge distributions and to electron charge shifts, ΔE_elstat_ and ΔE_orb_, are less important [2]. For the above-mentioned three complexes, −Δ**E_int_** does not exceed 3 kcal/mol, and in two cases it is close to 1 kcal/mol. The electron charge shifts for these complexes (Table 2) do not exceed 4 millielectrons! The latter is connected with the practically unchanged carbon charge in the CFH_3_ unit in those complexes in comparison with the isolated CFH_3_ molecule. For the remaining complexes electrostatic interaction energy is the most important attractive term, only in a case of the SiFH_3_-C_6_H_6_ and GeFH_3_-C_6_H_6_ complexes the dispersive term is slightly “less negative” than the electrostatic one. If one excludes the above-mentioned three CFH_3_ complexes, thus for majority of remaining complexes the orbital interaction, ΔE_orb_, is the next most important attractive term, after electrostatic interaction.

**Figure 3:**
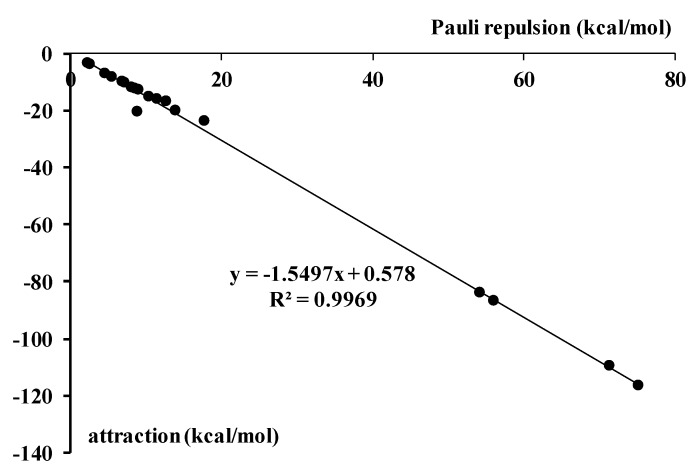
The linear correlation between the repulsion interaction energy and the sum of attractive terms (both in kcal/mol) for the ZFH_3_-B complexes analyzed here.

It was discussed in recent studies on hydrogen bonds and on other σ-hole bonds that these interactions are accompanied by effects that are a response for the Pauli repulsion [2,44]. The latter was also discussed for halogen bonds where multivalent halogen center plays a role of the Lewis acid while the π-electrons are the Lewis base [33]. For such interactions correlations were found between the repulsion interaction energy and different terms of the attractive interaction. It was found in earlier studies than the orbital interaction energy (if one refers to the decomposition scheme applied here) well correlates with the repulsion term, correlations for other interaction energy terms are not so good. However, in general, the sum of all attractive terms correlates with the Pauli repulsion term [2,33,44]. Figure 3 presents such a correlation for the complexes analyzed here. Thus the attractive interaction which is related to various effects related to complexation, among them to the electron charge redistribution, is a response for the Pauli repulsion.

**Figure 4:**
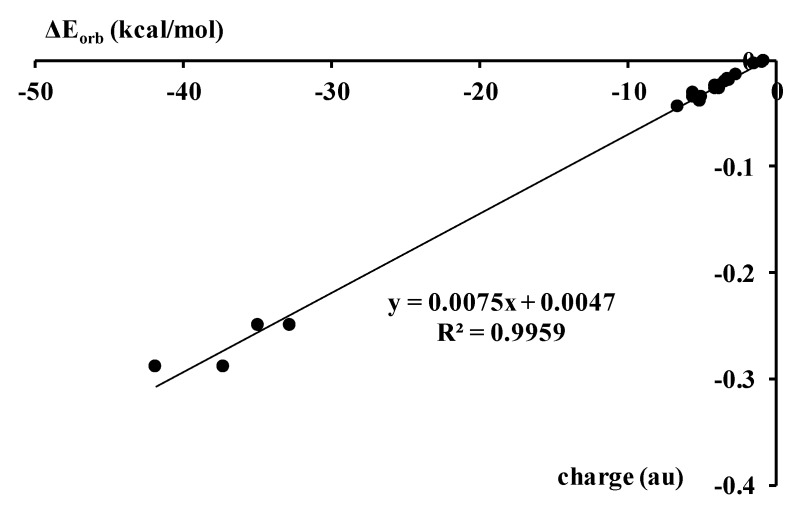
The linear correlation between the orbital interaction energy, ΔE_orb_, and the electron charge shift from the Lewis base unit to the Lewis acid (au).

The orbital interaction reflecting electron shifts corresponds to energy terms which are named in the other way in other decomposition schemes; most often they are labeled as the delocalization interaction energy, induction, charge transfer, polarization and others [2]. Figure 4 shows, for the complexes analyzed here, the correlation between the orbital energy, ΔE_orb_, and the electron charge shift resulting from complexation.

### 2.3. Quantum Theory of Atoms in Molecules Parameters

Table 4 presents characteristics of the bond critical point (BCP) of the bond path that connects the Lewis acid and Lewis base units of the complex. It is a link between the tetrel center (tetrel attractor) or the hydrogen center (hydrogen attractor) and the critical point of the Lewis base species. This critical point may correspond to the carbon atom attractor, to the bond critical point (BCP) of the CC bond or to the non-nuclear attractor (NNA) located on the CC bond path. Hence one can see that there are various topologies of complexes analyzed here. 

The above-mentioned bond path may concern the tetrel bond if the Z-center of the Lewis acid unit is linked with the Lewis base critical point or it may concern the hydrogen bond if the H-atom attractor of the Lewis acid is linked with the Lewis base critical point. One may expect the (Z)H···C bond paths show some “artificial interactions”, especially since the meaning of the bond path and its usefulness to analyze interactions is often a subject of controversies [45] and disputes [46,47]. The presented here preliminary results on tetrel bonds where π-electrons play a role of Lewis bases need additional extended studies. However few arguments that the accompanying (Z)H···C bond paths observed for some benzene and cyclopentadienyl complexes may correspond to weak hydrogen bonds are listed here. The electron densities at the (Z)H···C bond critical points (BCPs) are not meaningless and they are comparable sometimes with such values for the Z···C BCPs; see the GeFH_3_-C_6_H_6_ complex for example (Table 4). The PbFH_3_-C_5_H_5_^−^ complex is an example where the greatest electron density at the H···C BCP is observed since it amounts 0.015 au; note that for the medium in strength hydrogen bond in the water dimer the electron density at the H···O BCP is equal to 0.023 au (MP2/6-311++G(d,p) results [48]). Additionally the H···C intermolecular contacts correspond to the attractive electrostatic interactions since the carbon centers of the C_6_H_6_ and C_5_H_5_^−^ moieties are characterized by the negative electrostatic potentials (EPs) while the H-centers of the ZFH_3_ species by the positive EPs.

**Figure 5:**
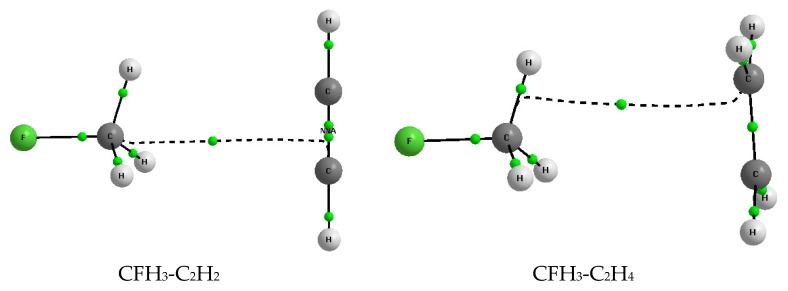
The molecular graphs of the CFH_3_-C_2_H_2_ and CFH_3_-C_2_H_4_ complexes; big circles—attractors, small green circles—BCPs, the nonnuclear attractor (NNA) is located (small red circle) between two BCPs in a case of the CFH_3_-C_2_H_2_ complex.

Particularly the following cases of bond paths are observed for complexes analyzed here. For the CFH_3_-C_2_H_2_ and CFH_3_-C_2_H_4_ complexes the irregular and nonlinear carbon-carbon bond paths are observed that may result from weak interactions (Figure 5); formally according to the QTAIM approach, they may be attributed to the tetrel bonds. For the CFH_3_-C_6_H_6_ (Figure 1) and CFH_3_-C_5_H_5_^−^ complexes the H···C intermolecular bond paths are observed which may be attributed to the C-H···C hydrogen bonds! For the SiFH_3_-C_2_H_2_, SiFH_3_-C_2_H_4_ and CFH_3_-C_6_H_6_ complexes the non-linear bond paths are detected, similarly as for the CFH_3_-C_2_H_2_ and CFH_3_-C_2_H_4_ complexes, which are attributed to the Si···C or H···C intermolecular links (Table 4).

In a case of the SiFH_3_-C_5_H_5_^−^ complex the clear almost linear Si···C bond path corresponding to the strong tetrel bond is observed, similarly as for the other ZFH_3_-C_5_H_5_^−^ complexes for Z = Ge, Sn and Pb. In a case of the PbFH_3_-C_5_H_5_^−^ complex the additional H···C bond path corresponding to the Pb-H···C hydrogen bond is observed (Figure 1). For the ZFH_3_-C_6_H_6_ complexes (Z = Ge, Sn, Pb) the tetrel and hydrogen bonds are observed with the corresponding bond paths, the SnFH_3_-C_6_H_6_ complex representing such a situation is presented in Figure 1. Similarly the SnFH_3_-C_2_H_2_, SnFH_3_-C_2_H_4_ complexes in Figure 1 reflect the same situation in analogues tin and lead complexes; in the case of acetylene Lewis base the Z···NNA bond path is observed while in the case of ethylene Lewis base this is the Z···BCP bond path.

The characteristics of bond critical points presented in Table 4 reflect the strength of interaction. It was discussed in various studies that these characteristics may be often treated as measures of the strength of interaction [49]; especially for homogeneous samples of complexes. Numerous relationships were found between characteristics of the H···B BCP and the strength of interaction for the A-H···B hydrogen bonds. For complexes analyzed here greater values of the electron density at the bond critical point, ρ_BCP_’s, are observed for the C_5_H_5_^−^ complexes. The Laplacian of the electron density at BCP, ∇^2^ρ_BCP_, is positive for all complexes analyzed which may show these are not covalent interactions; the H_BCP_ values are positive and close to zero for all complexes of acetylene, ethylene and benzene as well as for the CFH_3_-C_5_H_5_^−^ complex. For the remaining complexes of the C_5_H_5_^−^ anion the negative H_BCP_ values are observed that may indicate these are partly covalent in nature interactions.

One may ask what is the difference between the Z···π and Z···C tetrel bonds that are presented here. These “two kinds” of connections correspond to the types of bond paths. For the majority of acetylene and ethylene complexes former connections are observed while for the benzene and cyclopentadienyl complexes the latter ones. The Z···π bond path is a link between Z-attractor that corresponds to the nucleus and BCP or NNA located at the CC bond of acetylene or ethylene (see Figure 1). The Z···C bond path is a link between Z and C attractors corresponding to nuclei. This difference occurs within the Quantum Theory of Atoms in Molecules (QTAIM) scheme but it seems it is not observed in other approaches; for example in both cases the same orbital-orbital overlaps occur that correspond to the Z···π interactions; i.e., π_CC_ → σ_ZF_^*^ ones. All other accompanying overlaps specified earlier here are the same in both cases of contacts. The similar situations were observed earlier for the hydrogen bonded complexes with the π-electron systems playing a role of Lewis bases [50].

## 3. Computational Details 

The calculations were performed with the Gaussian16 set of codes [51] using the second-order Møller-Plesset perturbation theory method (MP2) [52], and the aug-cc-pVTZ basis set [53]. The relativistic effects for the heavier Ge, Sn and Pb atoms were taken into account. The calculations for these elements were done with quasi-relativistic small-core effective core potentials: ECP10MDF, ECP28MDF and ECP60MDF, for Ge, Sn and Pb, respectively [54]. For the latter elements the basis sets corresponding to aug-cc-pVTZ were applied, i.e., ECP10MDF_AVTZ, ECP28MDF_AVTZ and ECP60MDF_AVTZ, respectively [55]. Frequency calculations were performed for the complexes analyzed and their monomers to confirm that the optimized structures correspond to energetic minima. The binding energy, E_bin_, was calculated as difference between the energy of the complex and the sum of energies of monomers optimized separately while the interaction energy, E_int_, is a difference between the energy of the complex and the sum of energies of monomers which geometries come from the geometry of the complex considered [56]. The binding and interaction energies are negative but their difference—the deformation energy, E_def_ = E_bin_ − E_int_, is positive and it is connected with the change of geometries of monomers resulting from the complexation [39]. The Counterpoise (CP) correction was applied to calculate the basis set superposition error BSSE [57]; hence the E_bin_ and E_int_ values corrected for BSSE are analyzed in this study.

The Quantum Theory of ‘Atoms in Molecules’ (QTAIM) was also applied to characterize critical points (BCPs) in terms of the electron density (ρ_BCP_), its Laplacian (∇^2^ρ_BCP_) and the total electron energy density at BCP (H_BCP_) which is the sum of the potential electron energy density (V_BCP_) and the kinetic electron energy density (G_BCP_) [34]. The AIMAll program was used to carry out the QTAIM calculations [58]. 

The Natural Bond Orbital (NBO) method [35] was applied to calculate atomic charges, the electron charge shifts from the Lewis bases to the Lewis acids as well as the orbital-orbital interactions. The n_B_ → σ_AH_^*^ orbital-orbital interaction is characteristic for the A-H···B hydrogen bond; n_B_ labels the lone electron pair of the B Lewis base center and σ_AH_^*^ is the antibonding orbital of the A-H Lewis acid bond [35]. In a case of the hydrogen bonds where π-electrons and σ-electrons play a role of the Lewis bases, A-H···π and A-H···σ systems, the π_B_ → σ_AH_^*^ and σ_B_ → σ_AH_^*^ overlaps, respectively, are the most important orbital-orbital interactions [59]. The similar situation occurs for the tetrel bonds analyzed here, they may be classified as the Z···π or Z···C interactions (Z labels the tetrel centre). The π_CC_ → σ_ZF_^*^ and π_CC_ → σ_ZH_^*^ overlaps are observed here as the most important interactions; besides the σ_CH_ → σ_ZF_^*^ and σ_CH_ → σ_ZH_^*^ overlaps are also detected but they are characterized by lower energies than the former interactions. For example, the π_CC_ → σ_ZF_^*^ interaction is calculated as the second-order perturbation theory energy (Equation (1)):

ΔE (π_CC_ → σ_ZF_^*^) = −2 ⟨π_CC_|*F*|σ_ZF_^*^⟩^2^/(ε(σ_ZF_^*^) − ε (π_CC_)), (1)

⟨π_CC_|*F*|σ_ZF_^*^⟩ designates the Fock matrix element and (ε(σ_ZF_^*^) − ε (π_CC_)) is the orbital energy difference. The similar equations (to Equation (1)) for the remaining above-mentioned orbital-orbital interactions may be given. 

The energy decomposition analysis (EDA) [36,37] was carried out with the BP86 functional [60,61] in conjunction with the Grimme dispersion corrections (BP86-D3) [62] using uncontracted Slater-type orbitals (STOs) as basis functions for all elements with triple-ζ quality (ADF-basis set TZP). The energy decomposition analysis (EDA) was performed with the use of the ADF2013.01 program [63] for all complexes analyzed here and characterized by geometries resulting from the MP2/aug-cc-pVTZ optimizations. The EDA method follows the energy partition of Morokuma [36,37]. The interaction energy, ΔE_int_, between two fragments (A and B) in the A-B link, in the particular electronic reference state and in the frozen geometry of AB is considered in this approach. The ΔE_int_ interaction energy is divided into three components and the additional dispersion term, ΔE_disp_ (Equation (2)):

ΔE_int_ = ΔE_elstat_ + ΔE_Pauli_ + ΔE_orb_ + ΔE_disp_, (2)

The ΔE_elstat_ term corresponds to the electrostatic interaction between the unperturbed charge distributions of atoms and is usually attractive. The Pauli repulsion, ΔE_Pauli_, is the energy change associated with the transformation from the superposition of the unperturbed electron densities of the isolated fragments to the wavefunction which properly obeys the Pauli principle through explicit antisymmetrization and renormalization of the product wavefunction; it comprises the destabilizing interactions between electrons of the same spin on either fragment. The orbital interaction, ΔE_orb_, accounts for charge transfer and polarization effects.

**Figure 6:**
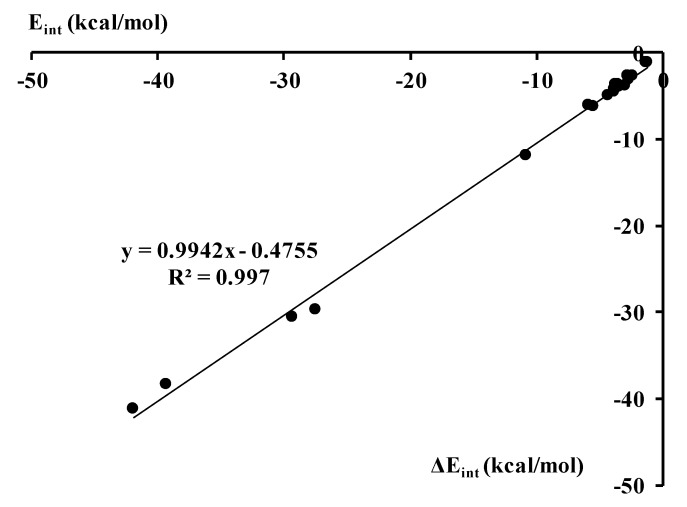
The linear correlation between the MP2 E_int_ interaction energy and the ΔE_int_ energy calculated within the DFT approach; both energies in kcal/mol.

Figure 6 presents the correlation between the interaction energy calculated within the MP2/aug-cc-pVTZ approach (Table 1), thus at the level corresponding to the systems’ optimizations, and ΔE_int_ DFT energy calculated with the use of ADF codes. The excellent correlation observed here partly justifies the use of DFT calculations for the previously optimized MP2 geometries. 

## 4. Conclusions and Perspectives

The tetrel bonds in complexes where the π-electron system plays a role of the Lewis base were analyzed here. Practically for all complexes considered the NBO approach shows the existence of the π_CC_ → σ_FZ_^*^ overlaps, however in a case of complexes of CFH_3_ with acetylene, ethylene and benzene the corresponding energies are negligible thus the existence of tetrel bonds is problematic. On the other hand for the remaining complexes the above-mentioned interactions are significant that may indicate the existence of the tetrel bonds. The QTAIM approach often shows the complicated topology, sometimes the additional bond paths corresponding to the hydrogen bonds are observed, or like for the CFH_3_-C_5_H_5_^−^ complex, only C-H···C intermolecular link is observed that may indicate the existence of the hydrogen bond and not of the tetrel bond. However for the other cyclopentadienyl complexes the interactions are very strong and the Z···C bond paths exist there. Hence there is no doubt that these complexes are linked by the tetrel bonds; all theoretical approaches applied in this study support the existence of such interactions in these complexes.

Only for some of acetylene and ethylene complexes one may observe the link between tetrel center and the site corresponding to π-electrons, NNA or BCP of the CC bond of the Lewis base unit (see the SnFH_3_-C_2_H_2_ and SnFH_3_-C_2_H_4_ complexes in Figure 1 as examples). In a case of the C_6_H_6_ and C_5_H_5_^-^ aromatic systems, the Z···C bond path is observed that suggest the one-atom Lewis base center and not the π-electron system. It means that the existence of two types of tetrel bonds may be considered within the QTAIM approach, Z···π and Z···C. However other approaches applied here do not distinguish rather between these types. Such a situation was earlier observed for the hydrogen bonded complexes [50]. 

**Figure 7:**
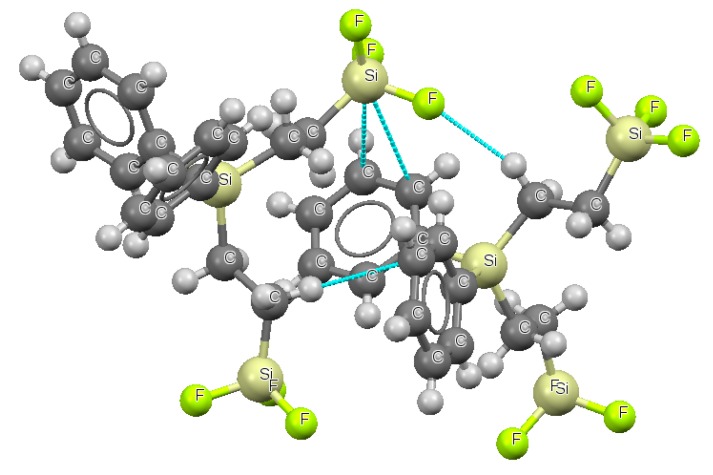
The fragment of the crystal structure (refcode: BAKZOF) where the Si···CC tetrel bond is observed.

The question arises if the interactions analyzed theoretically here really exist. This is why the Cambridge Structural Database (CSD) [64] search was performed. The following search criteria were taken into account; non-disordered structures, R less than 10%, 3D coordinated determined, non polymeric structures, single crystal structures and no errors (CSD updates up to February of 2018 were taken into account). The additional condition was that the Z tetrel center (C, Si, Ge, Sn and Pb) has to form two intermolecular Z···C contacts within corresponding sum of van der Waals radii. Two Z···C contacts were required since one may expect that in a case of double and triple CC bonds, or if CC bond concerns delocalized aromatic system; at least two Z···C distances within the van der Waals sum should be observed. 218 systems of crystal structures fulfilling those requirements were found in CSD. However only in 10 cases the clear tetrel-CC bond contacts with the tetrahedral (sp^3^ hybridized) tetrel center were observed which suggest the existence of the tetrel···π-electrons interactions. Figure 7 shows an example where one can observe the F-Si···CC contact (CC bond of the aromatic phenyl ring). This issue requires additional studies on the experimental crystal structures however. It seems that the search criteria could be also improved. More detailed study on experimental crystal structures’ results is in the progress.

## Figures and Tables

**Table 1:** The energetic parameters of complexes analyzed (in kcal/mol); interaction energy, E_int_, binding energy, E_bin_, deformation energy, E_def_ and BSSE correction. The distance between Lewis base and Lewis acid units is included—the shortest Z···C distance was chosen, the values in parentheses show if this distance is greater than the corresponding sum of van der Waals radii (positive values) or if it is lower (negative ones), distances in Å.

Complex	Distance	E_int_	E_bin_	E_def_	BSSE
CFH_3_-C_2_H_2_	3.428 (+0.53)	−1.3	−1.2	0.0	0.3
CFH_3_-C_2_H_4_	3.458 (+0.56)	−1.3	−1.3	0.0	0.4
CFH_3_-C_6_H_6_	3.398 (+0.50)	−2.7	−2.7	0.0	1.0
CFH_3_-C_5_H_5_^−^	3.241 (+0.34)	−10.8	−10.5	0.3	1.2
SiFH_3_-C_2_H_2_	3.344 (+0.04)	−2.4	−2.3	0.0	0.6
SiFH_3_-C_2_H_4_	3.315 (−0.02)	−2.7	−2.6	0.1	0.8
SiFH_3_-C_6_H_6_	3.253 (−0.05)	−3.7	−3.7	0.1	1.3
SiFH_3_-C_5_H_5_^-^	2.477 (−0.82)	−27.5	−18.1	9.4	2.0
GeFH_3_-C_2_H_2_	3.285 (−0.02)	−2.6	−2.6	0.1	1.1
GeFH_3_-C_2_H_4_	3.253 (−0.05)	−2.9	−2.9	0.1	1.5
GeFH_3_-C_6_H_6_	3.203 (−0.10)	−4.3	−4.2	0.1	2.7
GeFH_3_-C_5_H_5_^−^	2.525 (−0.78)	−29.3	−21.0	8.3	4.4
SnFH_3_-C_2_H_2_	3.325 (−0.13)	−3.4	−3.3	0.1	1.3
SnFH_3_-C_2_H_4_	3.280 (−0.17)	−3.8	−3.7	0.2	1.8
SnFH_3_-C_6_H_6_	3.183 (−0.27)	−5.5	−5.2	0.3	3.2
SnFH_3_-C_5_H_5_^−^	2.519 (−0.93)	−41.9	−30.6	11.4	5.2
PbFH_3_-C_2_H_2_	3.323 (−0.18)	−3.5	−3.4	0.1	2.2
PbFH_3_-C_2_H_4_	3.267 (−0.23)	−3.8	−3.7	0.1	3.1
PbFH_3_-C_6_H_6_	3.148 (−0.35)	−5.8	−5.6	0.3	5.7
PbFH_3_-C_5_H_5_^−^	2.624 (−0.88)	−39.3	−31.8	7.6	8.3

**Table 2:** The characteristics of complexes analyzed; ZF% and Angle% are the percentage increase of the Z-F distance and the percentage decrease of the F‒Z‒H angle, respectively; E_NBO_^1^ and E_NBO_^2^ are the NBO energies defined in the text (in kcal/mol); El-trans (au) is the electron charge transfer from the Lewis base to the Lewis acid while Z-charge is the charge of the Z-center in the complex considered (both charges in au calculated within NBO approach).

Complex	ZF%	Angle%	E_NBO_^1^	E_NBO_^2^	El-Trans	Z-Charge ^a^
CFH_3_-C_2_H_2_	0.14	0.00	0.5	0.0	−0.001	−0.158
CFH_3_-C_2_H_4_	0.14	0.00	0.6	0.0	−0.002	−0.158
CFH_3_-C_6_H_6_	0.29	0.09	0.4	0.8	−0.004	−0.156
CFH_3_-C_5_H_5_^-^	1.95	0.28	1.5	2.4	−0.030	−0.378
SiFH_3_-C_2_H_2_	0.25	0.65	1.7	0.3	−0.014	1.227
SiFH_3_-C_2_H_4_	0.31	0.74	2.3	0.4	−0.021	1.218
SiFH_3_-C_6_H_6_	0.31	0.74	1.8	1.0	−0.019	1.221
SiFH_3_-C_5_H_5_^−^	4.46	9.06	24.5	20.9	−0.250	1.086
GeFH_3_-C_2_H_2_	0.40	0.85	3.1	0.6	−0.019	1.025
GeFH_3_-C_2_H_4_	0.52	0.85	4.1	0.7	−0.027	1.015
GeFH_3_-C_6_H_6_	0.52	0.85	3.2	1.6	−0.025	1.021
GeFH_3_-C_5_H_5_^−^	5.18	8.95	33.4	22.1	−0.250	0.906
SnFH_3_-C_2_H_2_	0.46	1.34	4.1	1.2	−0.024	1.246
SnFH_3_-C_2_H_4_	0.57	1.43	5.4	1.9	−0.035	1.232
SnFH_3_-C_6_H_6_	0.62	1.72	4.8	2.9	−0.035	1.240
SnFH_3_-C_5_H_5_^−^	5.11	11.45	37.9	40.5	−0.289	1.148
PbFH_3_-C_2_H_2_	0.59	0.99	4.9	1.0	−0.027	1.085
PbFH_3_-C_2_H_4_	0.78	1.28	7.0	2.4	−0.039	1.070
PbFH_3_-C_6_H_6_	0.83	1.68	6.1	3.9	−0.044	1.080
PbFH_3_-C_5_H_5_^−^	5.44	8.78	36.5	31.0	−0.289	1.013

^a^ Z-charge for CFH_3_: −0.157, SiFH_3_: +1.240, GeFH_3_: +1.042, SnFH_3_: +1.267, PbFH_3_: +1.106 (all in au).

**Table 3:** The terms of the energy of interaction (kcal/mol); Pauli repulsion, ΔE_Pauli_, electrostatic, ΔE_elstat_, orbital, ΔE_orb_, dispersion, ΔE_disp_, and the total interaction energy, ΔE_int_.

Complex	ΔE_Pauli_	ΔE_elstat_	ΔE_orb_	ΔE_disp_	ΔE_int_
CFH_3_-C_2_H_2_	2.4	−1.3	−0.8	−1.4	−1.1
CFH_3_-C_2_H_4_	2.7	−1.4	−0.9	−1.6	−1.2
CFH_3_-C_6_H_6_	4.7	−2.4	−1.5	−3.6	−2.8
CFH_3_-C_5_H_5_^−^	8.9	−11.0	−5.6	−4.2	−11.9
SiFH_3_-C_2_H_2_	5.6	−3.4	−2.7	−2.2	−2.6
SiFH_3_-C_2_H_4_	7.3	−4.3	−3.5	−2.8	−3.2
SiFH_3_-C_6_H_6_	8.2	−4.1	−3.3	−4.5	−3.8
SiFH_3_-C_5_H_5_^−^	54.2	−43.4	−34.9	−5.6	−29.8
GeFH_3_-C_2_H_2_	7.1	−4.4	−3.2	−2.7	−3.2
GeFH_3_-C_2_H_4_	9.1	−5.5	−4.1	−3.4	−3.8
GeFH_3_-C_6_H_6_	10.6	−5.6	−4.1	−5.8	−5.0
GeFH_3_-C_5_H_5_^−^	56.1	−47.7	−32.8	−6.3	−30.7
SnFH_3_-C_2_H_2_	8.7	−5.8	−3.8	−3.0	−3.9
SnFH_3_-C_2_H_4_	11.6	−7.3	−5.0	−3.9	−4.6
SnFH_3_-C_6_H_6_	14.0	−7.8	−5.6	−6.9	−6.2
SnFH_3_-C_5_H_5_^−^	75.3	−68.1	−41.8	−6.6	−41.2
PbFH_3_-C_2_H_2_	9.2	−6.3	−3.8	−2.8	−3.6
PbFH_3_-C_2_H_4_	12.9	−8.3	−5.1	−3.7	−4.2
PbFH_3_-C_6_H_6_	17.9	−10.0	−6.6	−7.4	−6.2
PbFH_3_-C_5_H_5_^−^	71.4	−65.9	−37.2	−6.6	−38.4

**Table 4:** The QTAIM parameters (in au) of BCP of the Lewis acid—Lewis base bond path; electron density at BCP, ρ_BCP_, its laplacian, ∇^2^ρ_BCP_, and the total electron energy density at BCP, H_BCP_. The bond path type is also indicated.

Complex	ρ_BCP_	∇^2^ρ_BCP_	H_BCP_	BP-Type
CFH_3_-C_2_H_2_	0.005	0.020	0.001	C···C
CFH_3_-C_2_H_4_	0.005	0.018	0.001	C···C
CFH_3_-C_6_H_6_	0.007	0.022	0.001	(C)H···C
CFH_3_-C_5_H_5_^−^	0.011	0.038	0.001	(C)H···C
SiFH_3_-C_2_H_2_	0.007	0.022	0.001	Si···C
SiFH_3_-C_2_H_4_	0.008	0.023	0.001	(Si)H···C
SiFH_3_-C_6_H_6_	0.008	0.025	0.001	(Si)H···C
SiFH_3_-C_5_H_5_^−^	0.036	0.012	−0.012	Si···C
GeFH_3_-C_2_H_2_	0.009	0.027	0.001	Ge···NNA(CC)
GeFH_3_-C_2_H_4_	0.010	0.028	0.001	Ge···BCP(CC)
GeFH_3_-C_6_H_6_	0.010	0.028	0.001	Ge···C
-	0.007	0.024	0.001	(Ge)H···C
GeFH_3_-C_5_H_5_^−^	0.037	0.045	−0.008	Ge···C
SnFH_3_-C_2_H_2_	0.010	0.025	0.001	Sn···NNA(CC)
SnFH_3_-C_2_H_4_	0.011	0.029	0.001	Sn···BCP(CC)
SnFH_3_-C_6_H_6_	0.012	0.029	0.001	Sn···C
-	0.007	0.022	0.001	(Sn)H···C
SnFH_3_-C_5_H_5_^−^	0.045	0.066	−0.011	Sn···C
PbFH_3_-C_2_H_2_	0.011	0.034	0.001	Pb···NNA(CC)
PbFH_3_-C_2_H_4_	0.013	0.036	0.001	Pb···BCP(CC)
PbFH_3_-C_6_H_6_	0.014	0.039	0.001	Pb···C
-	0.009	0.026	0.001	(Pb)H···C
PbFH_3_-C_5_H_5_^−^	0.042	0.074	−0.008	Pb···C
-	0.015	0.041	0.001	(Pb)H···C
